# Dasatinib preferentially induces apoptosis by inhibiting Lyn kinase in nilotinib-resistant chronic myeloid leukemia cell line

**DOI:** 10.1186/1756-8722-4-32

**Published:** 2011-08-02

**Authors:** Seiichi Okabe, Tetsuzo Tauchi, Yuko Tanaka, Kazuma Ohyashiki

**Affiliations:** 1 First Department of Internal Medicine, Tokyo Medical University, 6-7-1 Nishi-shinjuku, Shinjuku-ku, Tokyo 160-0023, Japan

## Abstract

Nilotinib is approved for treatment of newly diagnosed chronic myeloid leukemia (CML) and it is shown superiority over imatinib in first-line treatment for patients of CML. In this study, we established a nilotinib-resistant cell line, K562NR, and evaluated the resistance to nilotinib and efficacy of dasatinib. We found activation of Lyn plays a dominant role in survival of the nilotinib-resistant cell line. We found dasatinib induces the apoptosis of nilotinib-resistant cells and inhibits Lyn kinase activity. This novel nilotinib-resistant CML cell line may help to explore novel therapy for CML.

## To the editor

**Figure 1:**
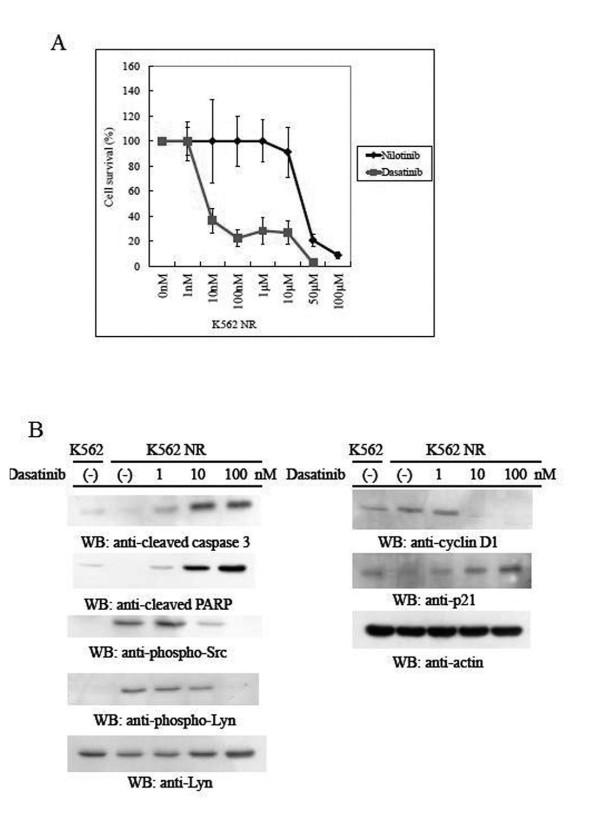
**Cell growth inhibition by dasatinib and cellular signaling in a nilotinib resistant cell line**. (A) K562NR cells exposed to dasatinib or nilotinib for 72-hrs were quantitated by cell proliferation. Each result is presented as the mean percentage of proliferation of unexposed control cultures. (B) Phosphorylation of Lyn, Src, cleaved caspase 3, PARP, p21, cyclin D1, and actin levels were analyzed by immunoblotting using the protein (30 μg) from cell lysates.

The BCR/ABL kinase inhibitor, imatinib, is the single effective and the standard treatment for chronic myeloid leukemia (CML) [1]. Resistance to imatinib is now a problem clinically. Imatinib resistance is often attributed to the emergence of clones expressing the BCR/ABL mutation and several other mechanisms such as overexpression of BCR/ABL and activation of Src-related kinase [2]. Nilotinib (AMN107) is a new BCR/ABL inhibitor and is highly selective for ABL kinase and 30-fold more potent than imatinib. Nilotinib has produced hematological and cytogenetic responses in CML patients, who did not initially respond to imatinib or developed imatinib resistance [3]. Recently, in Evaluating Nilotinib Efficacy and Safety in clinical Trials-newly diagnosed CML (ENESTnd), nilotinib has shown superior efficacy as front line treatment for patients with CML-chronic phase (CP) in comparison with imatinib [4,5]. Although nilotinib has shown superiority over imatinib in first-line treatment for CML-CP patients, the management of CML following the development of nilotinib resistance remains a challenge. In this study, we established a nilotinib-resistant cell line, K562NR, and evaluated the resistance to and efficacy of dasatinib. BCR/ABL levels were not increased by fluorescence in situ hybridization (FISH) analysis (data not shown). K562NR cells had no point mutation in Abl kinase (data not shown). K562 NR cells were resistant to high concentrations of nilotinib, with the IC50 being more than 10 μM (Figure 1A). Dasatinib (BMS-354825), a second generation tyrosine kinase inhibitor, is another promising new clinical candidate for CML treatment and has also shown good efficacy in CML patients, including imatinib-resistant cases. Dasatinib is an effective therapy after imatinib and nilotinib therapy failure in CML patients [6]. The phase III dasatinib versus imatinib study in treatment-naïve CML patients (DASISION) study demonstrates superior efficacy of dasatinib over imatinib and an acceptable safety profile [5,7]. We found that dasatinib reduced the cell growth of K562NR and significantly induced apoptosis. The IC50 of dasatinib is 5 nM (Figure 1A). We found that K562NR cells underwent increased phosphorylation of Src family kinase (SFK) including Lyn (Figure 1B). Phosphorylation of SFK was reduced after 24-hrs dasatinib treatment in a dose-dependent manner. Cleaved caspase 3 and poly (ADP-ribose) polymerase (PARP) were detected after 24-hrs dasatinib treatment (Figure 1B). We noted that protein levels of p21 increased and cyclin D1 was reduced after dasatinib treatment (Figure 1B). In our experiment, dasatinib also potentially induced apoptosis of the nilotinib-resistant cell line. Dasatinib was effective in 13 of the 23 patients with CML after imatinib and nilotinib therapy failure, including 7 patients who had a cytogenetic response [6]. These patients exhibited several Abl kinase mutations such as E255V/K. The resistance to imatinib in BCR/ABL positive cells has been reported to be associated with the activation of PI3K/AKT1 pathways [8]. In this study, there was no mutation in Abl kinase, but Src family kinases, including Lyn, was activated in the nilotinib-resistant cell line. Lyn kinase has been previously shown to be an important component in cytokine signal transduction, and is also reported to play a role in the growth and apoptotic regulation of hematopoietic cells [9]. Activation of SFK including Lyn may play a dominant role in the proliferation and survival of the nilotinib-resistant cell line, and the reduction of SFK phosphorylation may act at the p21 and cyclin D1 level and induce the apoptosis of K562NR cells after dasatinib treatment. This study showed that secondary signaling events involving SFK/Lyn in a nilotinib-resistant CML cell line may play a significant role for in the resistant mechanism.

## List of abbreviations

CML: chronic myeloid leukemia; CP: chronic phase; ENESTnd: evaluating nilotinib efficacy and safety in clinical trials-newly diagnosed CML; FISH: fluorescence in situ hybridization; DASISION: dasatinib versus imatinib study in treatment-naïve CML patients

## Conflicts of interests

The authors declare that they have no competing interests.

## Authors' contributions

SO performed the experimental procedures; TT, YT and KO designed and coordinated the study and interpreted data. All authors have read and approved the final manuscript.
